# Supplementary material for: The Diffusion Model of Intra-Golgi Transport Has Limited Power
Source: Int J Mol Sci. 2023 Jan 10;24(2):1375. doi: 10.3390/ijms24021375 (PMC9861033; doi:10.3390/ijms24021375)
Supplement: Supplementary file 1 [file ijms-24-01375-s001.zip › ijms-1931220-supplementary.pdf]

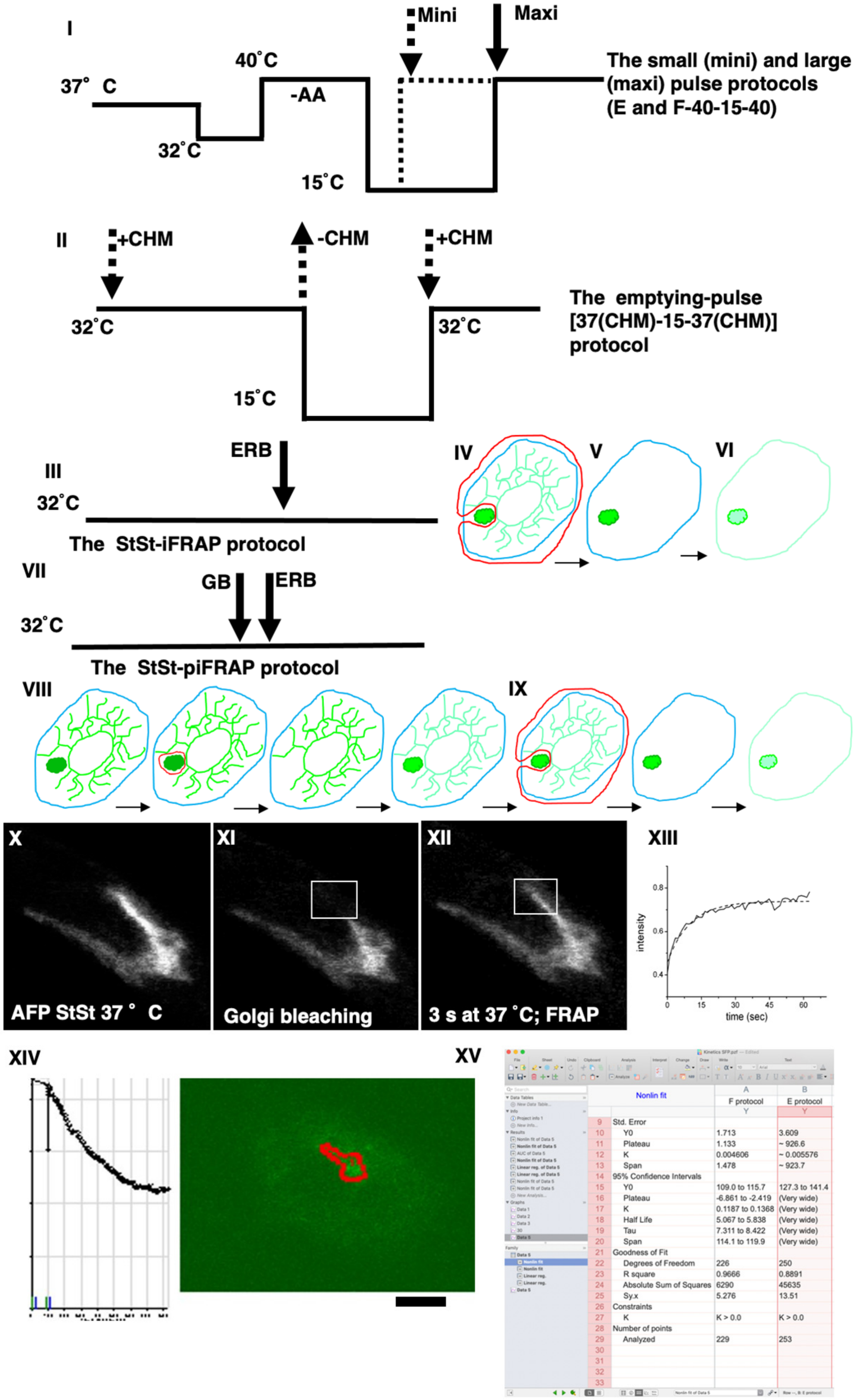

I
37° C
32°C
40°C
-AA
15°C
Mini
Maxi
The small (mini) and large (maxi) pulse protocols (E and F-40-15-40)
II
+CHM
-CHM
+CHM
32°C
15°C
32°C
The emptying-pulse [37(CHM)-15-37(CHM)] protocol
III
32°C
ERB
The StSt-iFRAP protocol
IV
V
VI
VII
32°C
GB
ERB
The StSt-piFRAP protocol
VIII
IX
X
AFP StSt 37 ° C
XI
Golgi bleaching
XII
3 s at 37 °C; FRAP
XIII
intensity
time (sec)
XIV
XV
Nonlin fit
F protocol
E protocol
Std. Error
Y0 1.713 3.609
Plateau 1.133 ~ 926.6
K 0.004606 ~ 0.005576
Span 1.478 ~ 923.7
95% Confidence Intervals
Y0 109.0 to 115.7 127.3 to 141.4
Plateau -6.861 to -2.419 (Very wide)
K 0.1187 to 0.1368 (Very wide)
Half Life 5.067 to 5.838 (Very wide)
Tau 7.311 to 8.422 (Very wide)
Span 114.1 to 119.9 (Very wide)
Goodness of Fit
Degrees of Freedom 226 250
R square 0.9666 0.8891
Absolute Sum of Squares 6290 45635
Sy.x 5.276 13.51
Constraints
K K > 0.0 K > 0.0
Number of points
Analyzed 229 253

Figure S1. Schemes demonstrating cargo synchronization protocols (I–IX). E-empty GC; F-filled GC; CHM, Cycloheximide; ERB, ER bleaching; GB, Golgi bleaching; FRAP, fluorescence recovery after photobleaching; arrows indicate time of the influence application (see Table 1). (I) The small (mini) and large (maxi) pulse protocols (E- and F-40-15-40). (II) The emptying-pulse [37(CHM)-15-37(CHM)] protocol. Arrows indicate the time when CHM was added and then eliminated. Horizontal line indicate temperature of the incubation medium. (III–VI) Scheme of iFRAP protocol. It includes bleaching of the whole cell less its GC (inside red curve) and measuring (arrows) of the fluorescence inside GZ (decrease of green colour intensity in green structure in [V] and [VI] and appearance of green colour in external line indicating PM). (VII–IX) Scheme of the piFRAP protocol. It includes bleaching of the Golgi area (small area inside red curve), refilling of this area bleaching of the whole cell less its GC (green structure with green borders) bleaching of the whole cell less its GC (inside red curve) and measuring (arrows) of the fluorescence inside GZ (decrease of green colour intensity in green structure in [IX] and appearance of green colour in external line indicating PM). (A–F) Analysis of the kinetics of the Golgi zone fluorescence using iFRAP at steady state (A–F) and piFRAP at steady state (G, H) synchrniozation protocols. (A) Initial position of cell. (E) Example of the image visdible on the microscopic screen. Decreasing of the GC fluorescence inside GC. (X–XII) The kymogram shows FRAP inside the white box in the cell transfected with AFP. In cells transfected with AFP FRAP is fast. (XIII) Data from plates X–XII are plotted. (XIV) Screen shot from the screen of the laser scanning confocal microscope shows how data for regression lines were acquired. Bleaching of the whole cell less its GC (outside red curve). (XV) Screen shot from the computer screen showing the estimation of the goodness-of-fit parameters. Scale bars: 1 μm (XI–XII); 5 μm (XIV).

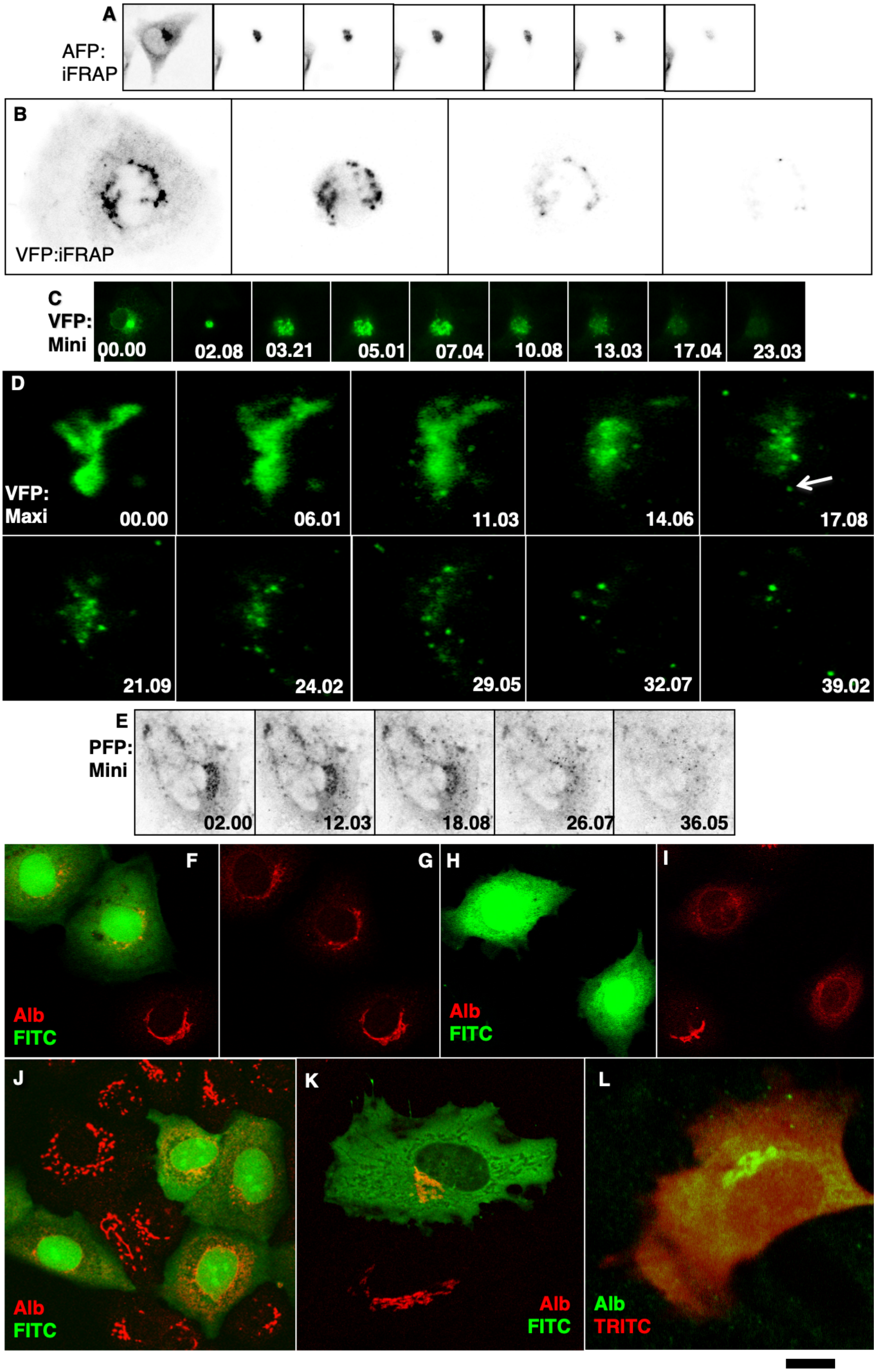
A
AFP:
iFRAP
B
VFP:iFRAP
C
VFP:
Mini
00.00
02.08
03.21
05.01
07.04
10.08
13.03
17.04
23.03
D
VFP:
Maxi
00.00
06.01
11.03
14.06
17.08
21.09
24.02
29.05
32.07
39.02
E
PFP:
Mini
02.00
12.03
18.08
26.07
36.05
F
Alb
FITC
G
H
Alb
FITC
I
J
Alb
FITC
K
Alb
FITC
L
Alb
TRITC

Figure S2. Representative additional examples of the kymograms describing the kinetic of the different cargo exit from the GZ using different synchronization protocols. (A) AFP:iFRAP. (B) VFP:iFRAP. (C) VFP:Mini-wave. (D) VFP:Maxi-wave. E); PFP:Mini-wave. The bleaching was not used (see Table 1). The names of cargo and the synchronization protocols are shown in panels. The movies were used for the measurement of the integral fluorescence within the GZ during IGT. (F, G and H, I) Two representative examples of experiments demonstrating Role of membrane fusion. Microinjection of α-SNAP mutant into HepG2 cells blocked entrance of albumin into the GC from the ER (B, D). (J) Microinjection (green cells: FITC labelling in [G–K]; red cells; TRITC labelling in [L]) of alpha-SNAP mutant into HepG2 cells inhibited exit of albumin (red in G–K) from the ER. (K, L) Microinjection the irrelevant protein (anti-albumin antibody) did not block exit of albumin (red in [K], green in [L]; TRITC red in [L)) from the ER. Scale bars: 7 μm (A,C); 2 μm (B, D); 5 μm (E–J); 2 μm (K, L).
